# Does reconstructing superficial branch of radial nerve defect with a split homolateral graft prevent painful neuromas?

**DOI:** 10.1186/s13018-026-06862-9

**Published:** 2026-04-16

**Authors:** Yingliang Liu, Xiahan Liu, Xu Zhang, Jingming Xie, Zhi Zhao, Yanjun Liu, Gang Li, Yingsong Wang

**Affiliations:** 1 Department of Orthopaedics, Second Affiliated Hospital of Kunming Medical University, No.374 Dianmian Road, Kunming, 650101 Yunnan China; 2 Department of Orthopaedics, People’s Hospital of Chuxiong Yi Autonomous Prefecture, Chuxiong, 675000 Yunnan China; 3 Department of Hand Surgery, Third Hospital of Hebei Medical University, Shijiazhuang, 050051 Hebei China; 4 Department of Medical Ultrasonics, People’s Hospital of Chuxiong Yi Autonomous Prefecture, Chuxiong, 675000 Yunnan China

**Keywords:** Reconstruction, Superficial branch of the radial nerve, Nerve defect, Nerve graft, Repair

## Abstract

**Objective:**

This study aimed to assess the efficiency of reconstructing the superficial branch of the radial nerve using a split homolateral graft to prevent painful neuromas.

**Methods:**

From January 2019 to October 2022, 32 patients (32 forearms) with traumatic defects of the superficial branch of the radial nerve were treated using a split homolateral graft. The assessments included pain, two-point discrimination, and patient satisfaction. For comparison, another 35 patients (35 forearms) who did not undergo nerve repair were reviewed.

**Results:**

The mean age of the nerve repair group was 36 years (range, 18–52 years). The mean defect length was 42 ± 18 mm. The mean follow-up period was 28.5 ± 4.7 months. The mean age of the non-nerve repair group was 35 years (range, 19–54 years). The mean defect length was 45 ± 22 mm. The mean follow-up period was 27.7 ± 5.8 months. There were no significant differences in patient age (*P* > 0.05), defect length (*P* > 0.05), and follow-up period (*P* > 0.05). At the final follow-up, the pain scales at the injury site for the two groups were 0 ± 1 cm *vs* 5 ± 4 cm (*P* < 0.05) based on the 10-cm visual analog scale. Sensation on the dorsum of the thumb were 4.8 ± 1.5 mm for the injured hand, 4.2 ± 1.4 mm for the opposite hand, and 9.3 ± 1.6 for the non-nerve repair hand (*P* < 0.05). Sensation on the first web were 5.2 ± 1.5 mm for the injured hand, 4.7 ± 1.2 mm for the opposite hand, and 8.9 ± 2.6 mm for the non-verve repair hand, (*P* < 0.05). Based on the Short Assessment of Patient Satisfaction, patient satisfaction of the nerve repair group and non-nerve repair group were 22.3 ± 3.5 and 18.6 ± 4.7, respectively (*P* < 0.05).

**Conclusions:**

Traumatic defects of the superficial branch of the radial nerve can be reconstructed using a split homolateral graft. Most sensations can be restored without causing morbidity. The surgery may reduce the incidence of painful neuroma formation.

**Level of evidence:**

Therapeutic study, Level IVa.

## Introduction

Traumatic defects of superficial branch of the radial nerve (SBRN) may occur in distal forearm injuries, leading to painful neuromas and sensory loss in the dorsum of the first web space and proximal portion of the lateral 3.5 digits. In addition, they may be accompanied by painful neuromas and sensory abnormalities [1]. However, due to a lack of available nerve grafts, preventing painful neuromas through nerve branch reconstruction is challenging [2].

**Fig. 1:**
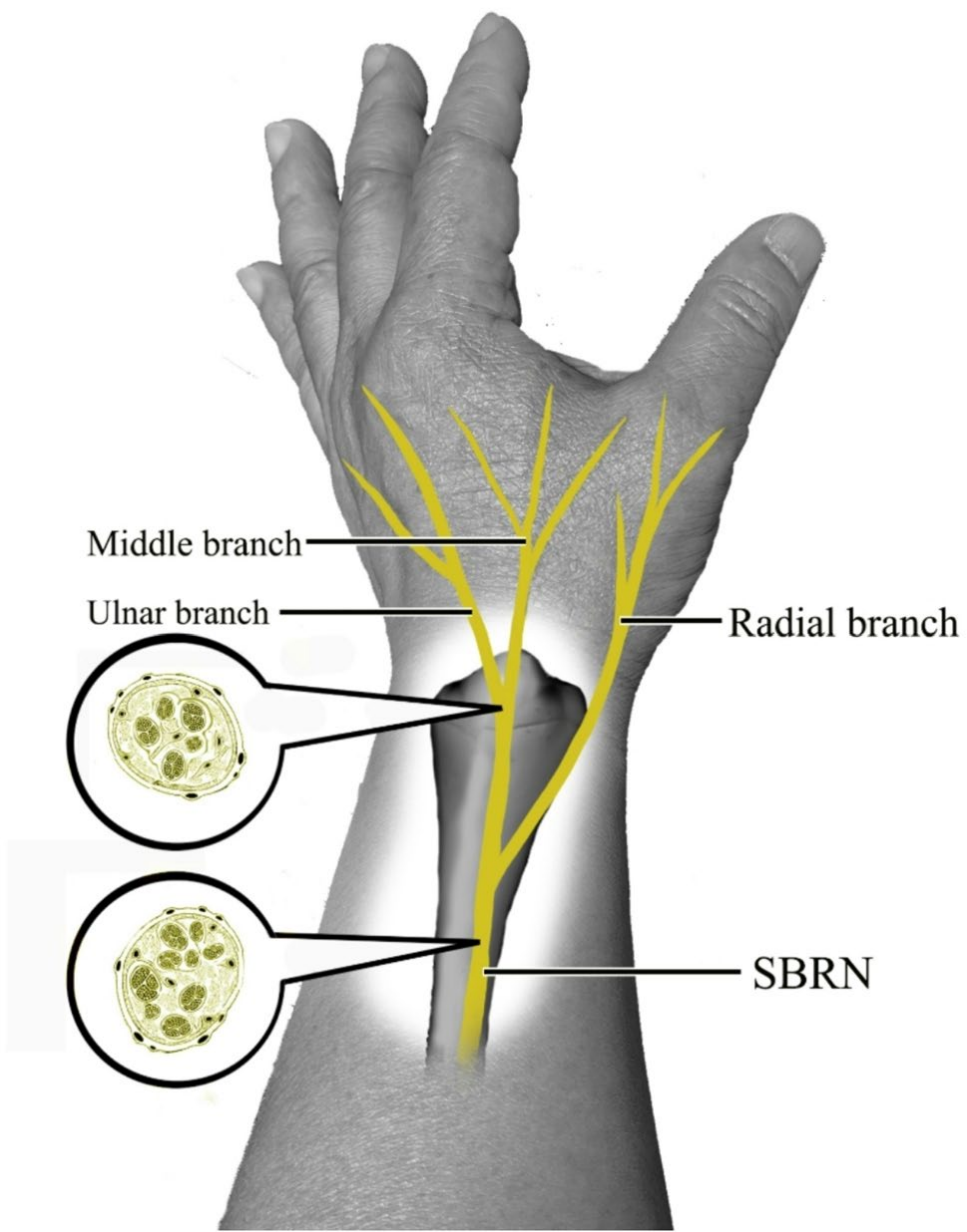
Anatomy of the superficial branch of the radial nerve (SBRN)

The SBRN runs along with the tendons of the brachioradialis muscle and extensor carpi radialis longus in the middle third part of the forearm [3]. The SBRN is a purely sensory branch of the radial nerve, providing innervation to the dorsolateral hand and dorsal surface of the thumb, index finger, and middle finger [4]. The SBRN has 3 (range, 2–6) main branches. The radial branch often originates about 4.5 cm (range, 2–7.9 cm) proximal to the radial styloid process (Fig. 1). The middle and ulnar branches usually bifurcate an average of 0.4 cm (range, 0–1.2 cm) proximal to the styloid process. These 3 branches contain an average of 4 (range, 2–7), 4.5 (range, 2–11), and 3.5 (range, 1–9) fascicles. However, the number and distribution patterns of these branches and fascicles vary widely [5].

Due to its partially superficial course, the SBRN is vulnerable to injury from trauma, potentially leading to painful neuroma [6]. If direct nerve repair is not possible, nerve grafting is only considered when sensory function recovery is crucial [7]. However, nerve grafting for the SBRN remains controversial because the sensory function of the dorsal hand is relatively less important [8]. Moreover, after nerve injury, disorganized regeneration of sensory axons can result in neuroma formation [9]. Painful neuromas can reduce patients' quality of life and place a burden on the healthcare system [10]. Surgeons are always aware of the trade-offs in sensory reconstruction. In this context, using the same divided nerve to reconstruct the SBRN is acceptable; it is a low-cost surgical method that does not cause donor site morbidities.

The objective of this retrospective study was to assess the efficiency of reconstructing the SBRN using a split homolateral graft in preventing painful neuromas. We also compared this surgical technique to non-nerve repair surgery.

## Patients and methods

This study was approved by the Ethics Review Committee of the Second Affiliated Hospital of Kunming Medical University in accordance with the principles expressed in the Declaration of Helsinki before it began. Written informed consent was obtained from each patient. Clinical trial number is not applicable. The data were obtained for research purposes after January 31, 2025.

From January 2019 to October 2022, consecutive patients with acute traumatic SBRN defects were included. Patients meeting the following inclusion criteria were eligible for the study: (1) aged between 18 and 55 years; (2) acute SBRN defects discovered during surgery that could not be directly repaired; (3) and patients who agreed to undergo nerve repair. Exclusion criteria were: (1) patients under 18 years were excluded due to the study focusing on adult neuropathy; (2) patients older than 55 years were excluded due to slower peripheral nerve regeneration; (3) old injuries; (4) patients without the normal opposite upper limb for comparison; (5) a history of nerve or peripheral vascular diseases; and (6) patients with diabetes, rheumatoid arthritis, or gout.

### Surgical technique

**Fig. 2:**
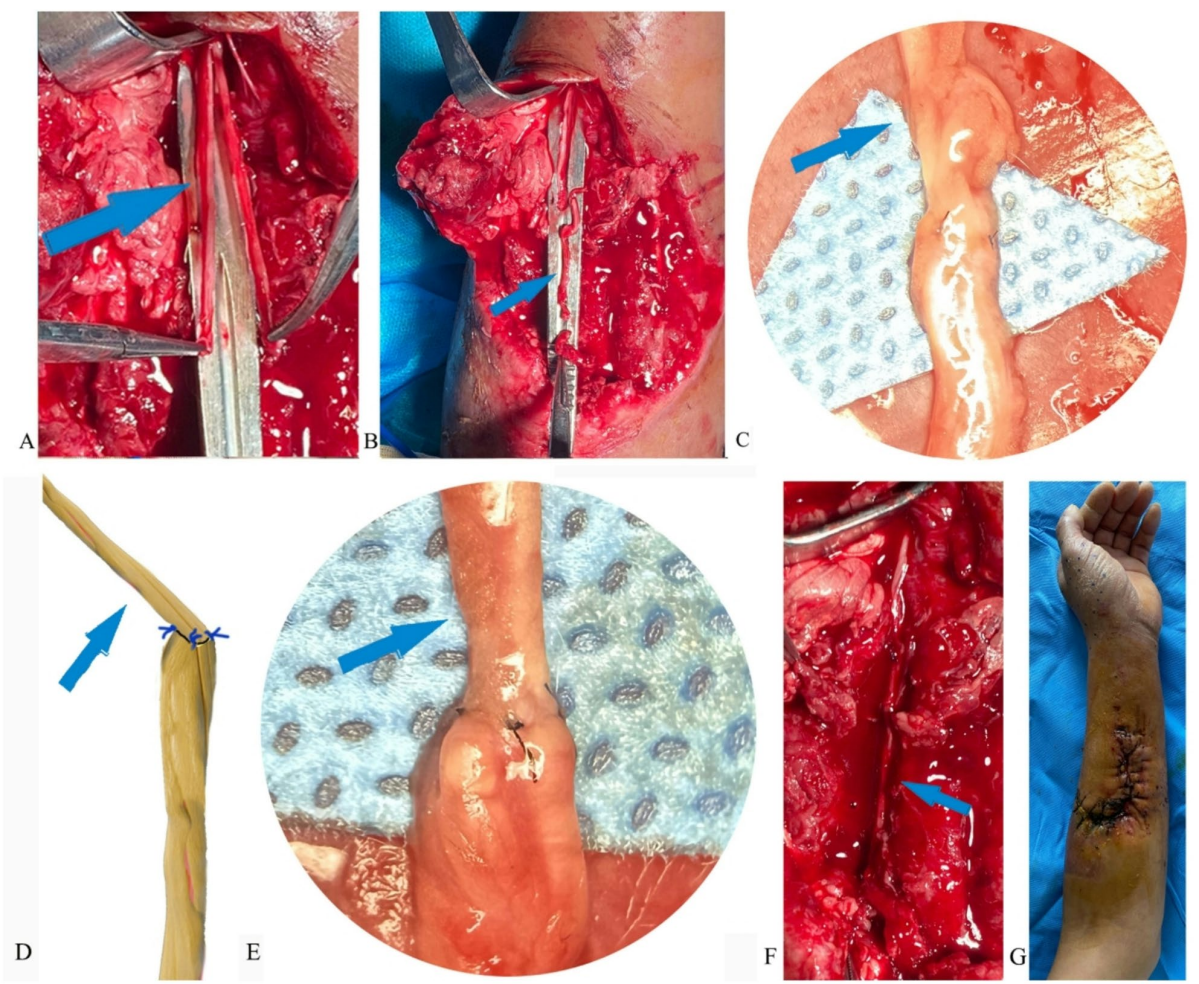
The SBRN defect is repaired using a split homolateral graft. **A** The distal SBRN is split, and the radial homolateral branch (arrow) is harvested as a nerve graft. **B** The SBRN defect is reconstructed using the nerve graft (arrow). **C** Under microscope, end-to-end neurorrhaphy is made between the distal graft end and distal end of the SBRN (arrow). **D** A diagram showing that the proximal graft end (arrow) is transected obliquely to increase the transverse-section area, followed by end-to-end neurorrhaphy between the small proximal graft end and large proximal end of the SBRN. **F** SBRN reconstruction is complete (arrow showing the nerve graft). **G** Wound appearance 10 days after surgery

The operation was performed under general anesthesia, intravenous regional anesthesia, or brachial plexus anesthesia, with the use of an upper arm tourniquet for control. After debridement, fractures, tendon injuries, vascular injuries, and nerve injuries were treated in the usual manner. The SBRN defect was assessed. The radial and middle branches were identified. The required length of the medial branch of SBRN (either distal or proximal segment depending on the injury) was split into a bundle for grafting (Fig. 2A, B). Using 9/0 ETHILON® nylon suture, end-to-end nerve coaptation was performed at both distal and proximal repair sites with the interposition nerve graft (Fig. 2C). At the proximal repair site, the graft end was transected obliquely so that the SBRN ends could be well coaptated to prevent neuroma formation in the future (Fig. 2D, E). Depending on the injury, the wound was closed directly or resurfaced with a flap (Fig. 2F, G). After surgery, a splint is used to protect the nerve repair site for 4 weeks, and range of motion exercises were initiated.

### Outcome evaluation

All assessments were conducted by an independent examiner who was not involved the treatments. Pain along the SBRN was assessed using the 10-cm visual analog scale [11]. The diagnosis of a neuroma was mainly based on persistent pain (> 1 month), with or without a positive Tinel sign at the injured SBRN site [12]. On ultrasound images, neuroma appeared as a well-circumscribed, hypoechoic lesion that was relatively mobile on direct pressure, [13]. We used measured hand sensory function using the two-point discrimination (2PD) [14]. The transverse diameter of SBRN was assessed using Aplio i800 Ultrasound system (Canon Medical Systems, Inc. USA) with a high-frequency transducer (7–14 MHz). Conduction velocity and response amplitude of the SBRN were assessed on the dorsum of the first web space [15]. Patient satisfaction was assessed using the Short Assessment of Patient Satisfaction [16].

### Statistical analysis

Quantitative variables that follow a normal distribution are presented as mean and standard deviation (SD), while variables with a non-normal distribution are presented as median and interquartile range. We used the chi-square test to analyze count data and selected appropriate analyses under different conditions. Rank data were analyzed using the Mann–Whitney U test, and measurement data were analyzed using one-way analysis of variance (One-Way ANOVA). Preoperative and postoperative control data at different time points for the two groups were analyzed using multivariate analysis of variance (multivariate ANOVA) and repeated measures analysis of variance (repeated measures ANOVA). A P value < 0.05 was considered statistically significant. All statistical analyses were performed using SPSS version 24.0 (IBM Corp., Armonk, NY, USA).

## Rusults

In this study, 32 patients (32 forearms) were selected from 38 consecutive with traumatic SBRN defects based on the inclusion criteria. We excluded 6 patients younger than 18 years (n = 3), older than 55 years (n = 4), and with a history of nerve injury (n = 1). The mean age of nerve repair group was 36 years (range, 18–52 years). The mean time from injury to surgery was 0 days (range, 0–1 day). The causes of injury included work-related (n = 24), road traffic accident, (n = 4) and other causes (n = 2). The mean defect length was 42 ± 18 mm. Three patients developed wound infections, which healed with wound care. All patients were followed up. The mean follow-up period was 28.5 ± 4.7 months (Table 1).

**Table 1:** Baseline demographics and clinical characteristics for 67 patients

	Nerve repair	Non-nerve repair	*t* value	P value
	(n = 32)	(n = 35)		
Age (mean; range; year)	36 (18–52)	35 (19–54)	1.35	0.13
Sex (m: f)	30: 2	31: 4	− 3	0.2
Dominant side (n)	17	16	− 6	0.66
Side affected (l: r)	17: 15	16: 19	− 3	0.66
Smoking (n)	4	6	− 3	0.2
Alcohol (n)	3	4	− 3	0.2
Injury to surgery (day)	0 (0–1)	0 (0–2)	− 1.3	0.23
Cause (n)				
Work	24	26	− 1.15	0.37
Road traffic accident	4	8		
Others	2	1		
Nerve defect (mean; range; mm)	42 ± 18	45 ± 22	2.24	0.13
Associations (n)				
Radius fracture	11	16	− 0.55	0.62
Tendon injury	15	12		
Vascular injury	5	4		
Major nerve injury	4	7		
Wound closure (n)				
Direct	24	24	− 2.3	0.26
Skin grafting	5	6		
Flap transfer	4	5		
Wound infection (n)	3	5	1.36	0.57
Follow-up (month)	28.5 ± 4.7	27.7 ± 5.8	− 0.78	0.22

For comparison, we reviewed another group of 35 patients (35 forearms) who were treated without nerve repair for traumatic SBRN defects. The mean age of non-nerve repair group was 35 years (range, 19–54 years). The mean time from injury to surgery was 0 days (range, 0–2 day). The causes of injury included work-related (n = 26), road traffic accident, (n = 8) and other causes (n = 1). The mean length of the defects was 45 ± 22 mm. Five patients developed wound infections, which healed with wound care. The mean follow-up period was 27.7 ± 5.8 months. There were no significant differences in patient age (*P* = 0.13), time from injury to surgery, nerve defect length (*P* = 0.13), or follow-up period (*P* = 0.22).

**Fig. 3:**
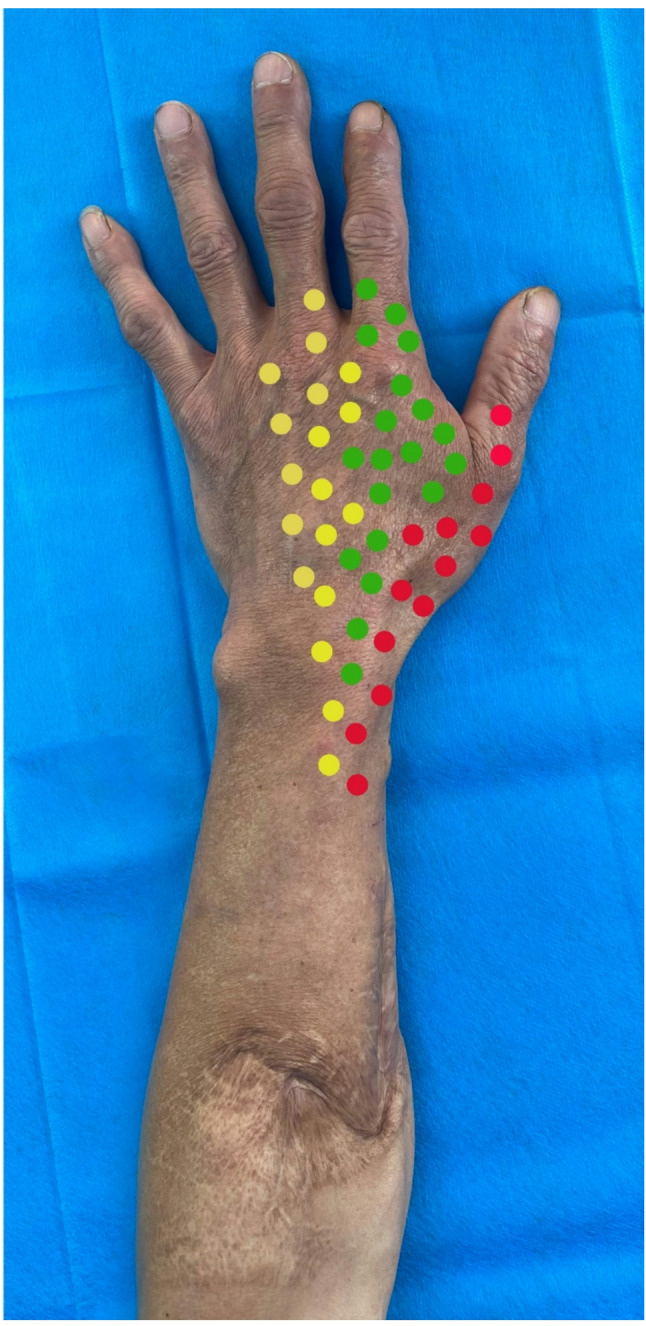
Assessment based on the sensory distribution of the SBRN (red dots, radial area; green dots, first web; and yellow dots, ulnar area)

**Fig. 4:**
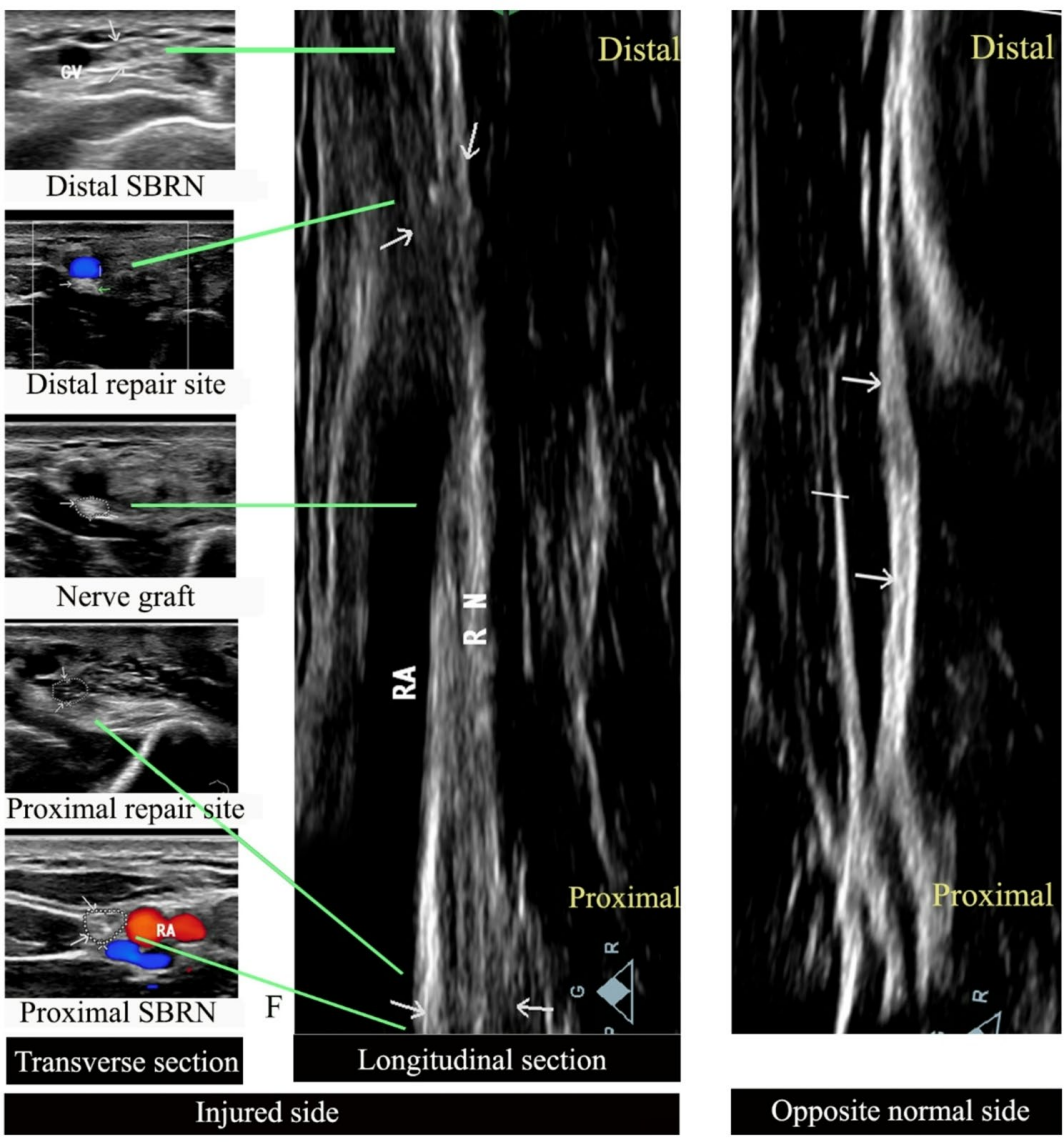
Ultrasonic images of the SBRN (also RN, arrows) at the final follow-up. The green lines show the levels of transverse sections corresponding to the axial section of the SBRN. RA, radial artery. CV, cephalic vein

At the final follow-up, neuromas occurred in 2 (6%) patients in the nerve repair group and 18 (51%) patients in the non-nerve repair group (< 0.01). Based on the 10-cm visual analog scale, pain scales at the injury site were 0 ± 1 cm *vs* 5 ± 4 cm for the two groups (*P* < 0.01) (Table 2). Sensation on the dorsum of the thumb were 4.8 ± 1.5 mm in the injured hand, 4.2 ± 1.4 mm in the opposite hand, and 9.3 ± 1.6 in the non-verve repair hand (P < 0.01) (Fig. 3). Sensation on the first web space were 5.2 ± 1.5 mm in the injured hand, 4.7 ± 1.2 mm in the opposite hand, and 8.9 ± 2.6 mm in the non-verve repair hand (P < 0.01). Conduction velocity of the SBRN was 45.6 ± 4.7 m/s in the nerve repair group and 0 ± 0 m/s in the non-nerve repair group (*P* < 0.01). The mean transverse diameter of the SBRN at the distal repair site was 1.7 ± 0.5 mm and 1.8 ± 0.9 mm, and at the proximal repair site was 1.8 ± 0.7 mm and 1.9 ± 0.9 mm (Tables 3 and 4; Fig. 4). Patient satisfaction for the nerve repair group and the non-nerve repair group were 22.3 ± 3.5 and 18.6 ± 4.7, respectively (*P* < 0.01).

**Table 2:** Outcomes of the treatments at the final follow-up

	Nerve repair	Non-nerve repair	*t* value	*P* value
	(n = 32)	(n = 35)		
Neuroma (n, %)	2 (6)	18 (51)	− 2.3	< 0.01
Pain (VAS; cm)	0 ± 1	5 ± 4	1.22	< 0.01
2-PD on thumb (mm)				
Injured side	4.8 ± 1.5	9.3 ± 1.6	− 1.66	< 0.01
Opposite side	4.2 ± 1.4	4.3 ± 1.2	− 2.32	0.36
*t* value	− 3.2	− 2.1		
P value	< 0.01	< 0.01		
2-PD on first web (mm)				
Injured side	5.2 ± 1.5	8.9 ± 2.6	− 1.87	< 0.01
Opposite side	4.7 ± 1.2	4.6 ± 0.9	− 2.35	0.75
*t* value	− 2.6	− 1.4		
P value	< 0.01	< 0.01		
2-PD on index finger (mm)				
Injured side	8.2 ± 1.5	8.3 ± 1.9	− 1.33	0.12
Opposite side	4.1 ± 1.6	4.2 ± 1.1	− 2.81	0.75
*t* value	2.36	− 1.35		
P value	< 0.01	< 0.01		
SBRN conduction (m/second)				
Injured side	45.6 ± 4.7	0 ± 0	2.66	< 0.01
Opposite side	62.3 ± 5.4	61.8 ± 5.7	1.22	0.27
*t* value	1.87	2.22		
P value	< 0.01	< 0.01		
Amplitude of response (microV)				
Injured side	32.2 ± 12.6	0 ± 0	1.17	< 0.01
Opposite side	42.2 ± 14.3	43.4 ± 13.5	2.24	0.14
*t* value	0.78	1.87		
P value	< 0.01	< 0.01		
Satisfaction (SAPS)	22.3 ± 3.5	18.6 ± 4.7	− 2.11	< 0.01

Data are shown as mean and standard deviation; VAS, visual analog scale score; 2PD, static two-point discrimination; SBRN, superficial branch of the radial nerve; SAPS, Short Assessment of Patient Satisfaction

**Table 3:** Ultrasonic transverse diameter of the SBRN of 32 patients at the final follow-up

	Injured forearm	Opposite forearm	%*	*t*	*P* value
2 cm distal to graft (mm)	1.6 ± 0.5	1.8 ± 0.7	89	1.85	< 0.01
Distal repair site (mm)	1.7 ± 0.5	1.8 ± 0.9	94	− 1.44	< 0.01
Middle point of graft (mm)	1.5 ± 0.6	1.9 ± 0.8	79	2.36	< 0.01
Proximal repair site (mm)	2.1 ± 1.1	1.9 ± 0.9	111	− 1.57	< 0.01
2 cm proximal to graft (mm)	1.8 ± 0.7	1.9 ± 0.9	95	2.33	0.24

Data are shown as mean and standard deviation; SBRN, superficial branch of the radial nerve; *, diameter of injured forearm/ diameter of opposite forearm

**Table 4:** Ultrasonic diameter of the superficial branch of the radial nerve of 35 patients

	Injured forearm	Opposite forearm	*t*	*P* value
Proximal end (mm)	4.6 ± 2.1	1.6 ± 0.8	− 2.33	< 0.01
2 cm proximal to proximal end (mm)	1.9 ± 0.8	1.8 ± 1	− 1.87	0.136

Data are shown as mean and standard deviation

## Discussion

Our study revealed that traumatic SBRN defects can be reconstructed using the split homolateral SBRN. The results show that partial sensation can be restored without causing morbidity, and it can yield good patient satisfaction. The diameter of the SBRN reaches at least 79% of the opposite normal nerve. The surgery may reduce the incidence of painful neuroma formation.

If primary repair of the SBRN cannot be performed without tension, nerve repair can be abandoned. However, its morbidity includes sensory loss on the dorsolateral skin of the hand and dorsum of the thumb, index finger, and long finger. Besmens et al. [10] reviewed 50 patients undergoing SBRN revision surgery and found that prolonged post-traumatic pain was a risk factor for persistent pain after surgical repair. After revision surgery, they found that all patients experienced persistent pain and suggested that early SBRN reconstruction may help prevent neuroma formation. In 1993, Brunelli et al. [17] described the muscle-in-vein technique, which involves filling a venous graft with an autologous muscle strip. Over the past 20 years, this technique has gradually been adopted as an effective method for bridging peripheral nerve defects. Ederer et al. [18] bridged 22 digital nerve defects (median 20 mm; range, 9–60 mm) with muscle-in-vein technique. Two years later, the median static and moving 2PD were 7 mm and 5 mm, respectively. However, in a systematic review and meta-analysis, Heinzel et al. [19] concluded that prognosis varied widely due to limited number of cases in different clinical settings. Currently, there is still no comprehensive systematic summary to determine efficiency and feasibility of muscle-in-vein conduits for reconstructing segmental nerve defects.

Traumatic neuromas typically present as a hard, oval, slowly growing, palpable nodule associated with pain, usually not exceeding 2 cm in diameter [20]. Common symptoms include pain, stiffness, pain hypersensitivity to light tactile stimuli, or neuralgic pain with a trigger point [21]. A neuroma is an abnormal, disorganized, spherical swelling formed at the severed end of a peripheral nerve, mainly composed of unmyelinated nerve endings, which occurs if timely repair is not performed [22]. Penna et al. [23] noted that patients with limb amputations are particularly susceptible, with incidences ranging from 4 to 48%. Regenerative peripheral nerve interface may be effective in treating and preventing neuroma formation. Woo et al. [24] found that among patients treated with regenerative peripheral nerve interfaces, 71% reported relief from neuroma pain, and 53% reported relief from phantom limb pain. Neurorrhaphy is another option for preventing neuromas formation, involving end-to-end or end-to-side coaptation of nerve ends through an epineural window [25]. However, this technique is limited by neural availability and requires a high level of technical difficulty, which may lead to unreliable results for less experienced surgeons [9]. Our surgical technique offers an alternative solution for restoring hand sensation and preventing the formation of painful neuromas. The outcomes may be superior to those reported in the literature.

The indication for using a split homolateral graft to reconstruct traumatic SBRN defects is that the homolateral graft is of sufficient length and the SBRN defect is located proximal to the level of the radial styloid. The advantage of this technique is that it can regenerate the SBRN with minimal trauma to the donor area. It seems that this technique does not have any significant disadvantages.

This study has limitations. It is difficult to demonstrate the specific contribution of homolateral SBRN graft to functional outcomes. The accurate assessment of sensory function is inherently subjective. Patients and surgeons are not fully blinded, and the surgeons' preferences, experience, and skills may influence the evaluation of the technique's effectiveness.

## Data Availability

Not applicable.
